# The disruption of GDP-fucose de novo biosynthesis suggests the presence of a novel fucose-containing glycoconjugate in *Plasmodium* asexual blood stages

**DOI:** 10.1038/srep37230

**Published:** 2016-11-16

**Authors:** Sílvia Sanz, Borja López-Gutiérrez, Giulia Bandini, Sebastian Damerow, Sabrina Absalon, Rhoel R. Dinglasan, John Samuelson, Luis Izquierdo

**Affiliations:** 1 ISGlobal, Barcelona Ctr. Int. Health Res. (CRESIB), Hospital Clínic - Universitat de Barcelona, Barcelona, Spain; 2 Department of Molecular and Cell Biology, Boston University Goldman School of Dental Medicine, 72 East Concord St, Boston, MA 02118, USA; 3 Division of Biological Chemistry and Drug Discovery, College of Life Sciences, University of Dundee, Dundee DD1 5EH, United Kingdom; 4 Division of Infectious Diseases, Boston Children’s Hospital and Harvard Medical School, Boston MA 02115, USA; 5 The University of Florida Emerging Pathogens Institute, Department of Infectious Diseases & Pathology, Gainesville FL 32611, USA

## Abstract

Glycosylation is an important posttranslational protein modification in all eukaryotes. Besides glycosylphosphatidylinositol (GPI) anchors and *N*-glycosylation, *O*-fucosylation has been recently reported in key sporozoite proteins of the malaria parasite. Previous analyses showed the presence of GDP-fucose (GDP-Fuc), the precursor for all fucosylation reactions, in the blood stages of *Plasmodium falciparum*. The GDP-Fuc *de novo* pathway, which requires the action of GDP-mannose 4,6-dehydratase (GMD) and GDP-L-fucose synthase (FS), is conserved in the parasite genome, but the importance of fucose metabolism for the parasite is unknown. To functionally characterize the pathway we generated a *PfGMD* mutant and analyzed its phenotype. Although the labelling by the fucose-binding *Ulex europaeus* agglutinin I (UEA-I) was completely abrogated, GDP-Fuc was still detected in the mutant. This unexpected result suggests the presence of an alternative mechanism for maintaining GDP-Fuc in the parasite. Furthermore, *PfGMD* null mutant exhibited normal growth and invasion rates, revealing that the GDP-Fuc *de novo* metabolic pathway is not essential for the development in culture of the malaria parasite during the asexual blood stages. Nonetheless, the function of this metabolic route and the GDP-Fuc pool that is generated during this stage may be important for gametocytogenesis and sporogonic development in the mosquito.

Protozoan parasites of the genus *Plasmodium* are the causative agents of human malaria, the most lethal being *Plasmodium falciparum*. Malaria is a global disease that presents symptoms ranging from fever and headaches to seizures, coma and death. Despite all the efforts to control malaria worldwide, 200 million malaria cases that led to more than half a million malaria deaths were reported in 2013^1^. Chemotherapy remains the most important tool for malaria control, although there are several vaccine candidates currently under evaluation^2^ and one of them has received regulatory approval^3^.

The life cycle of the parasite is highly complex, including development in two divergent hosts and in different tissue compartments. *Plasmodium* is transmitted to humans by the bite of infected female *Anopheles* mosquitoes. During a blood meal, sporozoites from the mosquito salivary glands are injected to humans and initiate infection in the liver. Parasites are then released from the liver into the bloodstream, where they invade red blood cells and reproduce asexually. A fraction (1–2%) of the parasites released from infected red blood cells develop into sexual stage gametocytes and are picked up by female *Anopheles* to initiate the sporogonic life cycle, resulting in the generation of sporozoite infection of mosquito salivary glands^4^.

Cell-surface glycoconjugates are important mediators of host-pathogen interactions for many microbes, including protozoan parasites^5^. Glycosylphosphatidylinositol (GPI) anchors are the major glycosylated molecules on the surface of the *Plasmodium* parasite^6^,^7^. Several GPI-anchored glycoproteins are essential for parasite invasion and virulence^8^ and parasite-derived GPIs, free or associated with protein, induce pro-inflammatory responses that contribute to the pathogenesis of malaria^9^,^10^. The presence of *N*-glycosylation of *P. falciparum* proteins has been a controversial issue for years^11^,^12^ that was recently solved by the characterization of very short *N*-glycans composed of one or two residues of *N*-acetylglucosamine (GlcNAc)^13^,^14^. However, the extent of protein *N*-glycosylation in the blood stages of the parasite seems to be rather low^15^. Furthermore, the existence of *O*-glycosylation and *C*-mannosylation in the parasite has been recently reported for the sporozoite stages^16^.

Sugar nucleotides are activated forms of sugars composed of a monosaccharide and a nucleotide moiety. These molecules are formed by either a salvage pathway, involving “activation” of the sugar using a kinase and a pyrophosphorylase, or by a *de novo* pathway, involving the bioconversion of an existing sugar or sugar nucleotide. These precursors feed the biosynthesis of glycans by acting as donors for glycosylation reactions^17^. We recently analysed the sugar nucleotide pool in asexuals and found that *P. falciparum* synthesises five different sugar nucleotides including GDP-fucose (GDP-Fuc) or UDP-galactose (UDP-Gal), both of which are not involved in the biosynthesis of the aforementioned GPI-anchors or *N*-glycans^18^. Therefore, the presence of these metabolites suggests that they may be involved in the biosynthesis of glycans not yet characterised in the parasite^19^. For example UDP-Gal may be required for the synthesis of the α-galactosyl epitopes recently identified in sporozoites^20^, which seem to be absent in the parasite asexual blood stages^21^. Similarly, GDP-Fuc could be involved in the posttranslational modification of thrombospondin type I repeat (TSR) domains present in several key proteins of the malaria parasite^16^,^22^.

In this work we analysed the functional role of the GDP-Fuc *de novo* biosynthetic pathway in the blood stages of *P. falciparum* by creating null mutants for GDP-mannose 4,6-dehydratase (GMD) and GDP-L-fucose synthase (FS). This metabolic route is not essential for the survival of the parasite in culture. Therefore we analysed the role of GDP-Fuc *de novo* biosynthesis by characterizing the phenotype of the null mutants *in vitro*.

## Materials and Methods

### Transfection constructs

**Figure 1:**
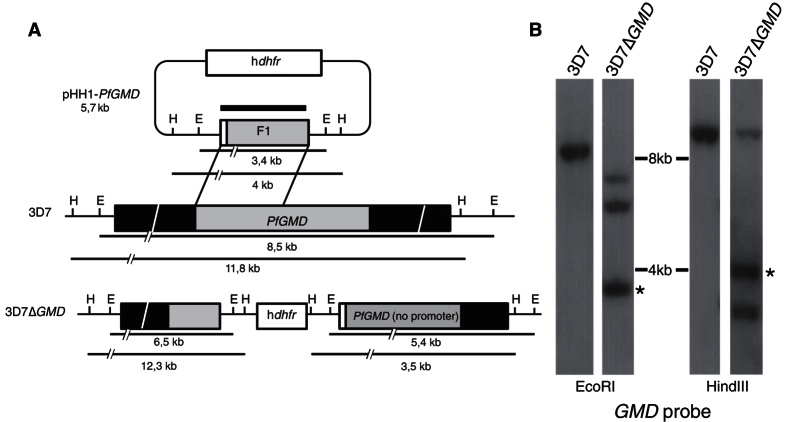
*PfGMD* transfection construct and integration events. (**A**) Schematic representation of the transfection plasmid (pHH1-*Pf*GMD) used to target and disrupt *GMD* gene in *P. falciparum* 3D7 parasites (3D7) and the expected recombination event (3D7∆*GMD*). The small white box in front of F1 fragment represents the absence of the first two ‘AT’ nucleotides in the start codon. The black boxes represent upstream and downstream DNA sequence flanking the *PfGMD* gene locus. The position of HindIII (H) and EcoRI (E) restriction sites, the position of *GMD* probe (thick black line) and the predicted length of the restriction fragments (thin black lines) are shown. (**B**) Southern Blot analysis of EcoRI and HindIII digested genomic DNA from 3D7 and 3D7Δ*GMD* parasites. Hybridisation of *GMD* probe to digested DNA from 3D7Δ*GMD* revealed restriction fragment sizes consistent with the disruption of *PfGMD* by the single recombination of the plasmid. *Matches with the expected size of the episomic plasmid copy. The panel shows a cropped blot and full-length blot is shown in Supplementary data (Supplementary Fig. S3).

To disrupt *Pf*GMD we generated a transfection construct based in the pHH1plasmid. pHH1-*Pf*GMD consisted of a ~500 bp sequence, from the start of the gene and without the first 2 nucleotides (AT), cloned into pHH1inv, a plasmid derived from pHH1 that excludes both promoter and termination sequences^23^,^24^. As a result of a single crossover homologous recombination event a disrupted copy of *GMD* and a copy without the first 2 nucleotides would be generated in parasites (Fig. 1A). The PCR product amplified using primers CGCGGTACCGCGAGTTGCTTTAATCTTCG and CACCCGCGGCCGCTTATATGATTCTCTATAATTTATTG was cloned into KpnI/NotI restriction sites (underlined) of plasmid pHH1inv.

To confirm our results, we also disrupted *Pf*FS using a pCC1-based construct. pCC1-*Pf*FS consisted of two fragments of ~1 kb and ~800 bp, respectively, from different regions of the *Pf*FS locus. F1 (nucleotides from −492 to +526 of the *Pf*FS locus) was amplified using primers ATCCCGCGGGTGTATAATATGTTGGTATAC and ATCACTAGTGTTAATGGTAGAGAACAATTTAC and cloned into SacII/SpeI restriction sites (underlined) of pCC1 plasmid^25^. Similarly, F2 (nucleotides 830 to 1584 of *Pf*FS locus) was amplified using primers AATCCCATGGACGAATGTATATCTTTTGGG and CTACCTAGGGATCTAATATCTTTATGAGATG and cloned into NcoI/AvrII restriction sites (underlined) of the same plasmid. The construct generated would integrate into *FS* disrupting it by double crossover homologous recombination (Supplementary Figure S1A)^25^.

### Southern blotting

Genomic DNA (gDNA) was isolated by Phenol/Chloroform method from ∼600 μl of red blood cells infected with >5% late trophozoites/schizonts. Two μg of 3D7- and 3D7ΔGMD-gDNA were digested with EcoRI and HindIII in separate reactions and probed with ^32^P (Perkin Elmer)-labelled *Pf*GMD. Similarly, 2 μg of 3D7-, SRΔFS- and 3D7ΔFS gDNA were digested with KpnI and HindIII or EcoRI and probed with ^32^P labelled F1 or F2 probes.

### Parasite culture and transfection

*P. falciparum* 3D7 (obtained from MR4-ATCC) parasites were cultured with human B^+^ erythrocytes (2–4% hematocrit) in RPMI medium (Sigma) supplemented with 10% AB^+^ human serum or 0.5% Albumax II, incubated at 37 °C in an atmosphere of 92% N_2_, 3% O_2_ and 5% CO_2_ using standard methods^26^. Human erythrocytes and serum were purchased from the Banc de Sang i Teixits (Catalonia, Spain), after approval from the Comitè Ètic Investigació Clínica Hospital Clínic de Barcelona. Parasite growth was monitored by counting the infected erythrocytes in Giemsa-stain blood smears by light microscopy. *P. falciparum* 3D7 parasites were transfected as described previously^23^. Briefly, 150 μg of each plasmid was used to electroporate (310 V, 950 millifarads) 200 μl of infected red blood cells at >5% parasitemia, synchronised for ring stage parasites. Transfected parasites were selected on 2 nM of WR99210 drug, and resistant parasites appeared in culture from 25 to 35 days after drug application. After the appearance of resistant parasites drug cycling with WR99210 was started. Clonal parasite lines were then derived from WR99210 resistant populations by limiting dilution. To calculate growth curves, tightly synchronised parasites were adjusted to 0.5% and measured by FACS. After 48 h and 96 h, parasitemia was again determined by FACS using SYTO 11 as previously described^27^.

### Heat-shock experiments

To measure survival to heat-shock^28^ parasites were sorbitol-synchronised and parasitemia adjusted to 1%. Heat shock was performed 22 h after sorbitol treatment by transferring cultures to an incubator at 41.5 °C for 3 h. Control cultures were maintained in a 37 °C incubator all the time. Parasitemia was determined by FACS at the next generation when all the schizonts had burst. Resistance to temperature stress was calculated by measuring the percentage of growth relative to identical cultures not subjected to heat-shock.

### Sugar nucleotide analysis

We synchronised the asexual cultures with sorbitol (or by combining Percoll and sorbitol treatments) and performed osmotic lysis of the trophozoite-infected red cells at ~35 h post-invasion and 10–12% parasitemia. Briefly, infected and uninfected erythrocyte pellets were resuspended twice in 60 volumes of erythrocyte lysis buffer (10× stock solution: 0.15 M NH^4^Cl, 0.1 M KHCO^3^, 0.01 M EDTA) and then incubated on ice for ~10 mins until lysis was completed (31). Pellets, consisting of free parasites and/or erythrocyte ghosts, were washed three times with cold phosphate-buffered saline (PBS), lysed in 70% ethanol in the presence of 20 pmols of GDP-glucose (GDP-Glc) internal standard, and sugar nucleotides were extracted using Envi-Carb (Supelco) columns^29^,^30^. Sugar nucleotides were then analysed using liquid chromatography tandem mass spectrometry (LC-MS/MS) and multiple-reaction monitoring (MRM) using a TSQ Quantiva Triple Quadrupole Mass Spectrometer (Thermo Scientific) or a QTRAP 6500 System (Sciex). A C18 reversed-phase column was used for sugar nucleotide separation with a gradient from 0.5% to 4% acetonitrile in 20 mM triethylammonium acetate buffer over 20 min at a flow rate of 25 µl/min. Sugar nucleotides were identified by their diagnostic MRM transitions^18^. The peak areas for each sugar nucleotide, along with their empirically determined molar relative response factors and the known amount of internal GDP-Glc, were used to quantify sugar nucleotides. Analyses were performed on three different sugar nucleotide extracts^30^.

### Uptake assays using tritiated sugars and GDP-Fuc

3D7 and *PfGMD* null mutant parasite cultures at 9–12% parasitemia were synchronized to the ring state and then washed and resuspended in RPMI medium supplemented with Albumax II. Cells were incubated at 3–4% hematocrit for 16–18 h, until the trophozoite stage. Cultures were then treated with saponin to release parasites and washed with glucose-free RPMI supplemented with Albumax II and 20 mM fructose. Free parasites were metabolically labeled with ^3^H-fucose (50 μCi/ml) and GDP-[^3^H]Fuc (30 μCi/ml) for 2 h, adapting the conditions used in previous works^31^. The same experiments were also repeated with infected RBCs not treated with saponin^32^. ^3^H-glucosamine and ^3^H-L-glucose were used as positive and negative control, respectively^32^,^33^. Triplicates of each condition, and duplicates in the case of GDP-[^3^H]Fuc, were included. Labeled parasites were washed three times with PBS at 4 °C and lysed, and the radioactivity was measured by liquid scintillation counting.

### Fluorescence microscopy

Cultured wild type and mutant *P. falciparum* lines were washed in PBS and fixed in 4% PFA and 0.075% glutaraldehyde in PBS overnight at 4 °C. Fixed cells were washed, permeabilised in 0.1% TX-100 in 1xPBS for 5 min at RT, washed again and then blocked in 3% BSA in 1xPBS overnight at 4 °C. All washes were performed in 0.5% BSA in 1xPBS by pelleting cells for 2 min at 800 g. Samples were incubated in solution 1 h at RT with ∼10 μg/ml *Ulex europaeus* agglutinin I (UEA-I) and 5 μg/ml *Griffonia simplicifolia* lectin II (GSL-II) conjugated to either Alexa Fluor488 or 594 (ThermoFisher Scientific). For the sugar inhibition experiments, the lectin solution was pre-incubated with 0.2 M methyl-α-fucopyranoside (αMeFuc) for 30 min at RT before being added to the cells. Nuclei were stained with 2 μg/ml DAPI (4,6-diaminido-2-phenylindole) for 15 min at RT following lectin incubation. Cells were washed in 0.5% BSA in 1xPBS, mounted using Vectashield (Vector Labs) and examined by deconvolving fluorescence microscopy using an Olympus IX70 microscope. Images were collected at 0.2-μm optical sections, deconvolved using SoftWoRx (Applied Precision) and further processed with Fiji^34^. Data is presented as a projection of the entire stack.

## Results

### Generation of *Pf*GMD (3D7Δ*GMD*) and *Pf*FS (3D7Δ*FS*) null mutants

To investigate the role of GDP-Fuc, we generated null mutants of the enzymes in the *de novo* biosynthetic pathway for this sugar nucleotide. For *Pf*GMD we used a targeting construct (pHH1-*Pf*GMD), which truncated the gene by single homologous recombination (Fig. 1A)^23^,^24^. In addition to the gene disruption, the single recombination event removed the first two nucleotides (AT) at the *Pf*GMD start codon. Thus, any translation products generated would be expected to yield aberrant or non-functional proteins. Southern blot analysis of restriction enzyme-digested DNA using a *Pf*GMD probe showed that the gene was mutated as intended (Fig. 1B). The isolation of 3D7Δ*GMD* mutants indicated that the gene was not essential. To confirm our results, we also disrupted the *Pf*FS locus using a double-crossover recombination approach based on a pCC1-targeting construct (pCC1-*Pf*FS) (Supplementary Fig. S1A)^25^. The disruption of both genes in the GDP-Fuc *de novo* pathway allow us to conclude that this metabolic route is not required for the survival of the blood stages of the parasite in culture.

### *PfGMD* null mutants did not exhibit a growth phenotype *in vitro*

**Figure 2:**
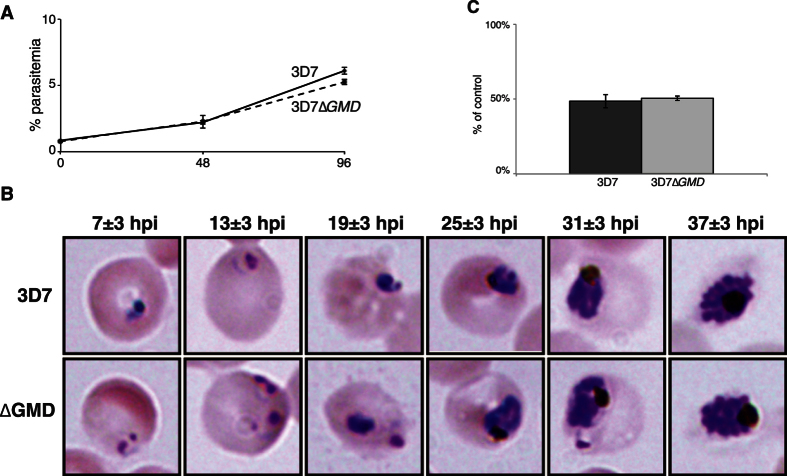
*P. falciparum* 3D7Δ*GMD* grow at comparable rates to 3D7 control cell lines. (**A**) Synchronous ring-stage 3D7Δ*GMD* and 3D7 growth was monitored over two complete life cycles (96h) by flow cytometry. (**B**) Intraerythrocytic development (x1000 magnification) of wild-type (3D7) and *Pf*GMD (ΔGMD) null mutants of *P. falciparum* 3D7 strain. Time indicates hours post-invasion of tightly (5 hours window) synchronised *in vitro* cultures by means of a combination of Percoll and sorbitol treatments. (**C**) Inhibition of 3D7 (dark grey bar) and 3D7Δ*GMD* (light grey bar) growth by a 3-h heat-shock at 41.5 °C. Values are the average of three independent replicas, with standard deviation, and represent percentage of growth relative to identical cultures not subjected to heat-shock. Heat-shock was performed when parasites were at the trophozoite stage and parasitemia was measured by FACS at the next generation. Statistical analysis, performed by applying one-way analysis of variance Tukey’s post-test, showed no significant differences.

3D7Δ*GMD* null mutant parasites showed growth rates similar to the 3D7 wild type strain (Fig. 2A), indicating that the GDP-Fuc *de novo* biosynthetic pathway is not necessary during *in vitro* blood stage development. In line with the above, we did not observe patent changes in cell size and morphology at the trophozoite stage between wild type and the mutant cell line (Fig. 2B).

Given that we did not observe a reduced growth phenotype for 3D7Δ*GMD* null mutants under *in vitro* conditions, we hypothesized that parasite survival may be impacted under stressful conditions. Glycosyltransferase activity and sugar nucleotides donors have long been linked to protein folding and thermotolerance in many organisms, including different parasites^35^,^36^,^37^. It has been suggested that Protein *O*-fucosyltransferase 2 (PoFUT2), of which a homolog is conserved and expressed in the *P. falciparum* genome^18^, may be involved in a novel quality control mechanism for the proper folding of TSR-domain containing proteins in the endoplasmic reticulum (ER)^37^,^38^. Proteins with TSR-domains have been shown to be expressed in asexual stages and to be involved in host-cell invasion^39^,^40^. To assess if abrogation of the GDP-Fuc *de novo* pathway could alter PoFUT2 function in the parasite, we studied the ability of 3D7Δ*GMD* mutant to cope with conditions likely to lead to the accumulation of misfolded TSR-containing proteins in the ER by measuring parasite growth after a 3 h heat shock at 41.5 °C. We did not observe any significant difference in resistance to heat shock in the mutant cell line compared to wild type, as judged by growth rate after temperature stress (Fig. 2C). Similar results were also observed for 3D7Δ*FS* cell line (Supplementary Fig. S1C).

### GDP-Fuc is still detected in the parasite after disruption of the GDP-Fuc *de novo* pathway

**Figure 3:**
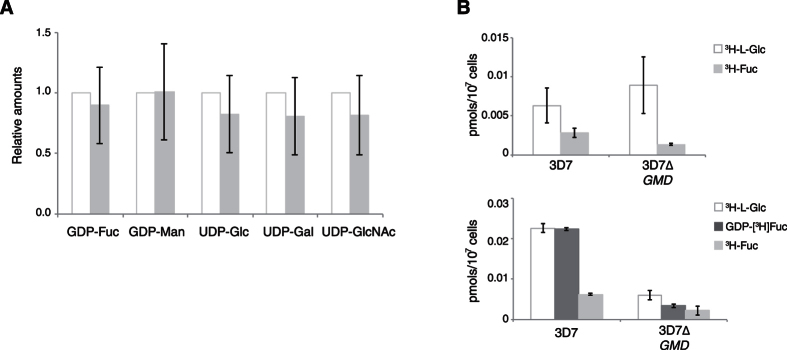
A pool of GDP-Fuc is detected in 3D7Δ*GMD* mutants. (**A**) The levels of GDP-Fuc and other sugar nucleotides are quantified in wild type (3D7; white bars) and 3D7Δ*GMD* mutants (grey bars). Values are shown as relative amounts to wild type sugar nucleotide levels and indicate the average variation ± S.D. of five different analyses. Analyses of every single experiment were always performed in triplicate. (**B**) Picomols of free ^3^H-fucose (light grey bars) and GDP-[^3^H]Fuc (dark grey bars) incorporated by wild type (3D7) and 3D7Δ*GMD* mutant parasites in culture (top) or after saponin treatment to release parasites (bottom). ^3^H-L-glucose (white bars) is included for comparison purposes, as it is incorporated through equilibrative (not active) transport mechanisms^45^. A control of ^3^H-glucosamine, incorporated by parasites^15^,^32^, was also used in every experiment (not shown).

To analyse the effect of *PfGMD* mutagenesis on parasite GDP-Fuc levels, sugar nucleotides from trophozoites were extracted, separated by reverse phase HPLC and quantified by multiple reaction monitoring tandem mass spectrometry using an internal standard (GDP-Glc) not present in *P. falciparum* blood stages^30^. We performed several biological replicates of the analyses, anticipating certain variability of the dynamic pools of sugar nucleotides. Nevertheless, the presence of GDP-Fuc in the mutant cell line was observed in all the experiments (Fig. 3A and Supplementary Table SI). Furthermore, the ratios between GDP-Fuc and other sugar nucleotides were similar throughout the different analyses, suggesting that there was not a specific reduction of the GDP-Fuc levels.

The detection of GDP-Fuc in the mutants strongly suggests the presence of an alternative pathway in the parasite for the generation of this sugar nucleotide. In order to identify an alternative mechanism of fucose transport and activation in *PfGMD* null mutants, we used tritiated sugar to perform uptake assays in infected RBCs or saponin-released parasites. However, no specific incorporation was observed in the *PfGMD* mutant cell line using either free fucose or GDP-Fuc (Fig. 3B).

### Fucose-binding lectin *Ulex europaeus* agglutinin 1 (UEA-I) labelling is abrogated in parasites with a disrupted GDP-Fuc *de novo* pathway

**Figure 4:**
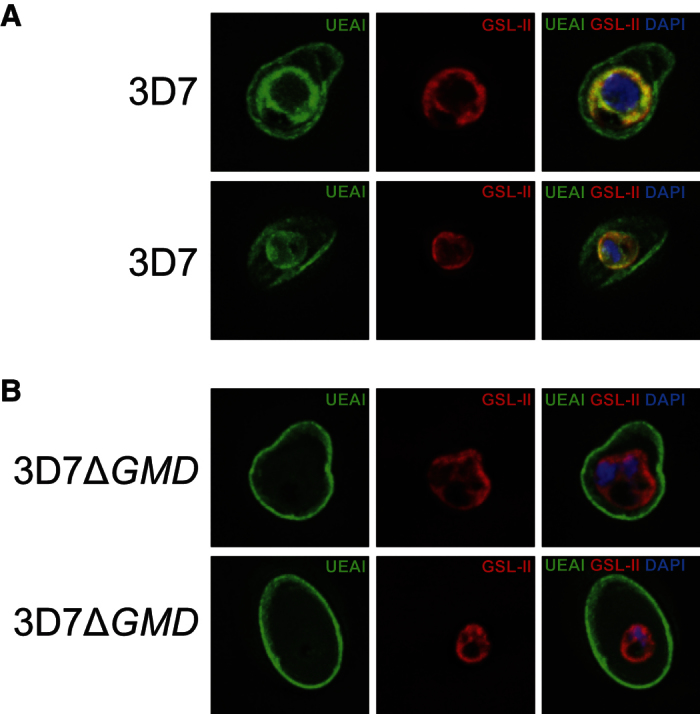
UEA-I labelling is abrogated in 3D7Δ*GMD* mutants. (**A**) Deconvolving micrographs of *P. falciparum* infected RBCs show a specific UEA-I labelling (green) on the surface of wild type parasites and host RBCs. GSL-II also binds to the surface of parasites^13^ (**B**) UEA-I labelling disappears in 3D7Δ*GMD* mutant surface (bottom panels) whereas GSL-II binding is not altered.

UEA-I, which has affinity for α-linked-fucose, binds to red blood cells (RBCs) membranes and also to wild type parasite surfaces (Fig. 4A). The UEA-I binding is absent in *PfGMD* null mutant parasites, suggesting that the mutation affects lectin binding to the surface of the parasite. On the contrary *Griffonia simplicifolia* lectin II (GSL-II) labelling, a GlcNAc-specific lectin which binds to *P. falciparum N*-glycans on the surface of the parasite and co-localises with UEA-I in wild type cells, is not affected in mutants (Fig. 4B)^13^. Therefore, GSL-II and UEA-I bind likely to different glycosylated structures on the plasma membrane of the parasite, but only UEA-I labelling is affected in mutants. UEA-I labelling specificity was confirmed by binding inhibition after lectin preincubation with αMeFuc (Supplementary Fig. S2).

## Discussion

Glycoconjugate biosynthesis requires the activation of monosaccharides to nucleotide sugars. These precursors are used by different glycosyltransferases for diverse glycosylation reactions^41^. Only five sugar nucleotides have been identified in the blood stages of *P. falciparum*: UDP-N-acetylglucosamine (UDP-GlcNAc), UDP-Gal, UDP-glucose (UDP-Glc), GDP-mannose (GDP-Man) and GDP-Fuc^18^. *P. falciparum* genome encodes and expresses homologues of GMD and FS, the enzymes responsible for the *de novo* biosynthesis of GDP-Fuc from GDP-Man^42^. Tritiated fucose is not taken-up significantly from the media by the parasite during the intraerythrocytic life cycle, which is consistent with the apparent absence of genes encoding for the GDP-Fuc salvage pathway in the parasite genome^18^,^43^,^44^. Altogether, this suggested that most, if not all, of the GDP-Fuc present is formed by the bioconversion of GDP-Man^18^. Surprisingly, the detection of GDP-Fuc in *PfGMD* mutants (Fig. 3A and Supplementary Table SI) implies that a substantial alternative source of GDP-Fuc is still present and active in the parasite. Our analyses with null mutant cell lines did not reveal the existence of an evident route for the incorporation of free fucose or GDP-Fuc (Fig. 3B). Nevertheless, it has to be considered that human erythrocytes contain significant levels of fucosylated proteins and their putative degradation may release monosaccharides that might be activated by the parasite. Furthermore, red blood cells also have a significant pool of GDP-Fuc (data not shown) which might be scavenged by the parasite during the intraerythrocytic life cycle. In this sense, previous studies had suggested that *P. falciparum* may obtain some sugars and/or other metabolites through passive mechanisms during the intraerythrocytic life cycle^45^,^46^.

A considerable variability in sugar nucleotide levels between different biological replicates was observed, independently of the synchronization method used (Fig. 3A). This disparity is possibly derived from inter-assay variability, differences on the sources of RBCs for *in vitro* culture or even fluctuations of the dynamic pools of sugar nucleotides in parasites. Nevertheless, GDP-Fuc was always detected and there was not a specific reduction of this sugar nucleotide in comparison to the rest of the pools, suggesting that the GDP-Fuc *de novo* pathway may not represent a significant contribution to the levels of GDP-Fuc in the blood stages of the parasite. Furthermore, mutant cell lines did not present alterations in their growth *in vitro* (Fig. 2A and Supplementary Fig. S1B). This also seems to be the case in *P. berghei* Δ*GMD* and Δ*FS* mutants^47^. Further studies may help to completely dissect the impact of the disruption of the GDP-Fuc *de novo* route in other stages of the parasite life cycle.

Nevertheless, fluorescence microscopy assays using AlexaFluor488-labelled UEA-I and performed on detergent-permeabilised and parasitised erythrocytes showed a striking reduction in lectin binding to the parasite membrane in *PfGMD* null mutant (Fig. 4B). Despite the presence of GDP-Fuc in the mutant, this reduction in UEA-I binding to a fucose glycotope might somehow be related to the disruption of a source of GDP-Fuc, namely the *de novo* pathway. If this metabolic route contributes even in part to the metabolite pool, mutants could be reacting to a marginal decrease of GDP-Fuc by shutting down the biosynthesis of fucose-containing glycans dispensable for *in vitro* growth (Fig. 4B). Examples of glycan biosynthesis cessation as adaptation to a lower availability of precursors have been reported recently^48^.

The decrease of UEA-I binding strongly suggests the presence of a fucose-containing glycan on the surface of *P. falciparum* blood stages. Despite several attempts, we were not successful in reproducing the observed differences by Western blot (not shown) perhaps indicating that UEA-I binds to a glycolipid in the parasites. UEA-I is best known for its ability to agglutinate human erythrocytes by binding to the α-1,2-fucose-contaning H-antigen^49^. Only erythrocytes of the rare Bombay phenotype, which are completely devoid of such epitopes due to an inactivated mutation in the α-1,2-fucosyltransferase gene (FUT1), are not agglutinated^50^,^51^. The reduction in UEA-I binding in mutant parasites indicates that a fucosyl transferase may be involved in the synthesis of a UEA-I-glycotope on the surface of the parasite.

No putative α-1,2-fucosyltransferase can be identified in the *P. falciparum* genome by bioinformatic searches, however a homolog of PoFUT2 (PF3D7_0909200) is present. PoFUT2 is involved in the *O*-fucosylation of TSR-domains in other organisms^38^. Peptides belonging to this putative *Pf*PoFUT2 have been detected in the sporozoite stages of the parasite^52^, when large amounts of TSR-containing proteins such as the circumsporozoite protein (CSP) and thrombospondin-related adhesive protein (TRAP) are expressed and posttranslationally modified^16^. Hence, there is the possibility that the *de novo* GDP-Fuc disruption results in a more striking phenotype during sporozoite development in the mosquito, potentially affecting both salivary gland and hepatocyte invasion. *P. falciparum* PoFUT2 gene is also upregulated during the schizont stages of the intraerythrocytic life cycle^18^ and therefore it may be involved in the fucosylation of other TSR-domain containing proteins required for host-cell invasion in these stages^16^,^39^,^40^,^53^. Although *Pf*PoFUT2 might be the fucosyltransferase involved in the biosynthesis of the UEA-I glycotope, there is also the possibility that other GDP-Fuc-dependent transferases are present and active throughout the blood stages of *P. falciparum*. Further work is being carried out at present to characterise this glycan modification and the glycosyl transferase/s involved in its biosynthesis.

## Additional Information

**How to cite this article**: Sanz, S. *et al.* The disruption of GDP-fucose de novo biosynthesis suggests the presence of a novel fucose-containing glycoconjugate in *Plasmodium* asexual blood stages. *Sci. Rep.* **6**, 37230; doi: 10.1038/srep37230 (2016).

**Publisher’s note:** Springer Nature remains neutral with regard to jurisdictional claims in published maps and institutional affiliations.

## Supplementary Material

Supplementary Information

## References

[b1] WHO. World Malaria Report 2014. *WHO* (2016).

[b2] Schwartz L., Brown G. V., Genton B. & Moorthy V. S. A review of malaria vaccine clinical projects based on the WHO rainbow table. Malar. J. 11, 11 (2012). 22230255 10.1186/1475-2875-11-11 PMC3286401

[b3] RTS,S Clinical Trials Partnership *et al.* Efficacy and safety of RTS,S/AS01 malaria vaccine with or without a booster dose in infants and children in Africa: final results of a phase 3, individually randomised, controlled trial. Lancet (London, England) 386, 31–45 (2015). 10.1016/S0140-6736(15)60721-8 PMC5626001 25913272

[b4] Kooij T. W., Janse C. J. & Waters A. P. Plasmodium post-genomics: better the bug you know? Nat Rev Microbiol 4, 344–357 (2006). 16582929 10.1038/nrmicro1392

[b5] Rodrigues J. A. *et al.* Parasite glycobiology: a bittersweet symphony. PLoS Pathog. 11, e1005169 (2015). 26562305 10.1371/journal.ppat.1005169 PMC4642930

[b6] McConville M. J. & Ferguson M. A. The structure, biosynthesis and function of glycosylated phosphatidylinositols in the parasitic protozoa and higher eukaryotes. Biochem. J. 294 (Pt 2), 305–324 (1993). 8373346 10.1042/bj2940305 PMC1134455

[b7] von Itzstein M., Plebanski M., Cooke B. M. & Coppel R. L. Hot, sweet and sticky: the glycobiology of Plasmodium falciparum. Trends Parasitol 24, 210–218 (2008). 18420458 10.1016/j.pt.2008.02.007

[b8] Sanders P. R. *et al.* A set of glycosylphosphatidyl inositol-anchored membrane proteins of Plasmodium falciparum is refractory to genetic deletion. Infect Immun 74, 4330–4338 (2006). 16790807 10.1128/IAI.00054-06 PMC1489731

[b9] Schofield L., Hewitt M. C., Evans K., Siomos M.-A. & Seeberger P. H. Synthetic GPI as a candidate anti-toxic vaccine in a model of malaria. Nature 418, 785–789 (2002). 12181569 10.1038/nature00937

[b10] Krishnegowda G. *et al.* Induction of proinflammatory responses in macrophages by the glycosylphosphatidylinositols of Plasmodium falciparum: cell signaling receptors, glycosylphosphatidylinositol (GPI) structural requirement, and regulation of GPI activity. J. Biol. Chem. 280, 8606–8616 (2005). 15623512 10.1074/jbc.M413541200 PMC4984258

[b11] Gowda D. & Davidson E. Protein glycosylation in the malaria parasite. Parasitol. Today 15, 147–152 (1999). 10322336 10.1016/s0169-4758(99)01412-x

[b12] Kimura E. A., Couto A. S., Peres V. J., Casal O. L. & Katzin A. M. N-linked glycoproteins are related to schizogony of the intraerythrocytic stage in Plasmodium falciparum. J Biol Chem 271, 14452–14461 (1996). 8662869 10.1074/jbc.271.24.14452

[b13] Bushkin G. G. *et al.* Suggestive evidence for darwinian selection against asparagine-linked glycans of Plasmodium falciparum and Toxoplasma gondii. Eukaryot Cell 9, 228–241 (2010). 19783771 10.1128/EC.00197-09 PMC2823003

[b14] Samuelson J. & Robbins P. W. Effects of N-glycan precursor length diversity on quality control of protein folding and on protein glycosylation. Semin. Cell Dev. Biol. 41, 121–128 (2014). 25475176 10.1016/j.semcdb.2014.11.008 PMC4452448

[b15] Gowda D. C., Gupta P. & Davidson E. A. Glycosylphosphatidylinositol anchors represent the major carbohydrate modification in proteins of intraerythrocytic stage Plasmodium falciparum. J Biol Chem 272, 6428–6439 (1997). 9045667 10.1074/jbc.272.10.6428

[b16] Swearingen K. E. *et al.* Interrogating the Plasmodium sporozoite surface: identification of surface-exposed proteins and demonstration of glycosylation on CSP and TRAP by mass spectrometry-based proteomics. PLoS Pathog. 12, e1005606 (2016). 27128092 10.1371/journal.ppat.1005606 PMC4851412

[b17] Caputto R., Leloir L. F., Cardini C. E. & Paladini A. C. Isolation of the coenzyme of the galactose phosphate-glucose phosphate transformation. J. Biol. Chem. 184, 333–350 (1950). 15422002

[b18] Sanz S. *et al.* Biosynthesis of GDP-fucose and other sugar nucleotides in the blood stages of Plasmodium falciparum. J. Biol. Chem. 288, 16506–16517 (2013). 23615908 10.1074/jbc.M112.439828 PMC3675586

[b19] Cova M., Rodrigues J. A., Smith T. K. & Izquierdo L. Sugar activation and glycosylation in Plasmodium. Malar. J. 14, 427 (2015). 26520586 10.1186/s12936-015-0949-z PMC4628283

[b20] Yilmaz B. *et al.* Gut microbiota elicits a protective immune response against malaria transmission. Cell 159, 1277–1289 (2014). 25480293 10.1016/j.cell.2014.10.053 PMC4261137

[b21] Ramasamy R. & Field M. C. Terminal galactosylation of glycoconjugates in Plasmodium falciparum asexual blood stages and Trypanosoma brucei bloodstream trypomastigotes. Exp. Parasitol. 130, 314–320 (2012). 22406352 10.1016/j.exppara.2012.02.017

[b22] Doud M. B. *et al.* Unexpected fold in the circumsporozoite protein target of malaria vaccines. Proc. Natl. Acad. Sci. USA. 109, 7817–7822 (2012). 22547819 10.1073/pnas.1205737109 PMC3356675

[b23] Crabb B. S. *et al.* Transfection of the human malaria parasite Plasmodium falciparum. Methods Mol Biol 270, 263–276 (2004). 15153633 10.1385/1-59259-793-9:263

[b24] Cortes A. *et al.* Epigenetic silencing of Plasmodium falciparum genes linked to erythrocyte invasion. PLoS Pathog 3, e107 (2007). 17676953 10.1371/journal.ppat.0030107 PMC1937010

[b25] Maier A. G., Braks J. A., Waters A. P. & Cowman A. F. Negative selection using yeast cytosine deaminase/uracil phosphoribosyl transferase in Plasmodium falciparum for targeted gene deletion by double crossover recombination. Mol Biochem Parasitol 150, 118–121 (2006). 16901558 10.1016/j.molbiopara.2006.06.014

[b26] Trager W. & Jensen J. B. Human malaria parasites in continuous culture. Science 193, 673–675 (1976). 781840 10.1126/science.781840

[b27] Urbán P., Estelrich J., Cortés A. & Fernàndez-Busquets X. A nanovector with complete discrimination for targeted delivery to Plasmodium falciparum-infected versus non-infected red blood cells *in vitro*. J. Control. Release 151, 202–211 (2011). 21223986 10.1016/j.jconrel.2011.01.001

[b28] Rovira-Graells N. *et al.* Transcriptional variation in the malaria parasite Plasmodium falciparum. Genome Res 22, 925–938 (2012). 22415456 10.1101/gr.129692.111 PMC3337437

[b29] Rabina J. *et al.* Analysis of nucleotide sugars from cell lysates by ion-pair solid-phase extraction and reversed-phase high-performance liquid chromatography. Glycoconj J 18, 799–805 (2001). 12441669 10.1023/a:1021107602535

[b30] Turnock D. C. & Ferguson M. A. Sugar nucleotide pools of Trypanosoma brucei, Trypanosoma cruzi, and Leishmania major. Eukaryot Cell 6, 1450–1463 (2007). 17557881 10.1128/EC.00175-07 PMC1951125

[b31] Wang P., Wang Q., Sims P. F. G. & Hyde J. E. Characterisation of exogenous folate transport in Plasmodium falciparum. Mol. Biochem. Parasitol. 154, 40–51 (2007). 17509698 10.1016/j.molbiopara.2007.04.002 PMC1906846

[b32] Gerold P., Dieckmann-Schuppert A. & Schwarz R. T. Glycosylphosphatidylinositols synthesized by asexual erythrocytic stages of the malarial parasite, Plasmodium falciparum. Candidates for plasmodial glycosylphosphatidylinositol membrane anchor precursors and pathogenicity factors. J Biol Chem 269, 2597–2606 (1994). 8300589

[b33] Woodrow C. J., Penny J. I. & Krishna S. Intraerythrocytic Plasmodium falciparum expresses a high affinity facilitative hexose transporter. J. Biol. Chem. 274, 7272–7277 (1999). 10066789 10.1074/jbc.274.11.7272

[b34] Schindelin J. *et al.* Fiji: an open-source platform for biological-image analysis. Nat. Methods 9, 676–682 (2012). 22743772 10.1038/nmeth.2019 PMC3855844

[b35] Izquierdo L., Atrih A., Rodrigues J. A., Jones D. C. & Ferguson M. A. Trypanosoma brucei UDP-glucose:glycoprotein glucosyltransferase has unusual substrate specificity and protects the parasite from stress. Eukaryot Cell 8, 230–240 (2009). 19114500 10.1128/EC.00361-08 PMC2643610

[b36] Naderer T., Wee E. & McConville M. J. Role of hexosamine biosynthesis in Leishmania growth and virulence. Mol. Microbiol. 69, 858–869 (2008). 18532982 10.1111/j.1365-2958.2008.06314.x

[b37] Vasudevan D. & Haltiwanger R. S. Novel roles for O-linked glycans in protein folding. Glycoconj. J. 31, 417–426 (2014). 25186198 10.1007/s10719-014-9556-4 PMC4214879

[b38] Luo Y., Koles K., Vorndam W., Haltiwanger R. S. & Panin V. M. Protein O-fucosyltransferase 2 adds O-fucose to thrombospondin type 1 repeats. J Biol Chem 281, 9393–9399 (2006). 16464857 10.1074/jbc.M511975200

[b39] Chattopadhyay R. *et al.* PfSPATR, a Plasmodium falciparum protein containing an altered thrombospondin type I repeat domain is expressed at several stages of the parasite life cycle and is the target of inhibitory antibodies. J. Biol. Chem. 278, 25977–25981 (2003). 12716913 10.1074/jbc.M300865200

[b40] Thompson J. *et al.* PTRAMP; a conserved Plasmodium thrombospondin-related apical merozoite protein. Mol. Biochem. Parasitol. 134, 225–232 (2004). 15003842 10.1016/j.molbiopara.2003.12.003

[b41] Coutinho P. M., Deleury E., Davies G. J. & Henrissat B. An evolving hierarchical family classification for glycosyltransferases. J Mol Biol 328, 307–317 (2003). 12691742 10.1016/s0022-2836(03)00307-3

[b42] Becker D. J. & Lowe J. B. Fucose: biosynthesis and biological function in mammals. Glycobiology 13, 41R–53R (2003). 10.1093/glycob/cwg054 12651883

[b43] Ginsburg H. Progress in in silico functional genomics: the malaria Metabolic Pathways database. Trends Parasitol 22, 238–240 (2006). 16707276 10.1016/j.pt.2006.04.008

[b44] Aurrecoechea C. *et al.* PlasmoDB: a functional genomic database for malaria parasites. Nucleic Acids Res 37, D539–D543 (2009). 18957442 10.1093/nar/gkn814 PMC2686598

[b45] Kirk K., Horner H. A. & Kirk J. Glucose uptake in Plasmodium falciparum-infected erythrocytes is an equilibrative not an active process. Mol. Biochem. Parasitol. 82, 195–205 (1996). 8946385 10.1016/0166-6851(96)02734-x

[b46] Downie M. J., Saliba K. J., Howitt S. M., Broer S. & Kirk K. Transport of nucleosides across the Plasmodium falciparum parasite plasma membrane has characteristics of PfENT1. Mol. Microbiol. 60, 738–748 (2006). 16629674 10.1111/j.1365-2958.2006.05125.x

[b47] Gomes A. R. *et al.* A genome-scale vector resource enables high-throughput reverse genetic screening in a malaria parasite. Cell Host Microbe 17, 404–413 (2015). 25732065 10.1016/j.chom.2015.01.014 PMC4362957

[b48] Damerow S. *et al.* Depletion of UDP-glucose and UDP-galactose using a degron system leads to growth cessation of leishmania major. PLoS Negl. Trop. Dis. 9, e0004205 (2015). 26529232 10.1371/journal.pntd.0004205 PMC4631452

[b49] Matsumoto I. & Osawa T. Purification and characterization of an anti-H(O) phytohemagglutinin of Ulex europeus. Biochim. Biophys. Acta - Protein Struct. 194, 180–189 (1969). 10.1016/0005-2795(69)90193-7 5353123

[b50] Kelly R. J. *et al.* Molecular basis for H blood group deficiency in Bombay (Oh) and para-Bombay individuals. Proc. Natl. Acad. Sci. 91, 5843–5847 (1994). 7912436 10.1073/pnas.91.13.5843 PMC44093

[b51] Le Pendu J., Cartron J. P., Lemieux R. U. & Oriol R. The presence of at least two different H-blood-group-related beta-D-gal alpha-2-L-fucosyltransferases in human serum and the genetics of blood group H substances. Am. J. Hum. Genet. 37, 749–760 (1985). 9556663 PMC1684624

[b52] Lindner S. E. *et al.* Total and putative surface proteomics of malaria parasite salivary gland sporozoites. Mol. Cell. Proteomics 12, 1127–1143 (2013). 23325771 10.1074/mcp.M112.024505 PMC3650326

[b53] Uchime O. *et al.* Analysis of the conformation and function of the Plasmodium falciparum merozoite proteins MTRAP and PTRAMP. Eukaryot. Cell 11, 615–625 (2012). 22467743 10.1128/EC.00039-12 PMC3346429

