# Supplementary material for: The disruption of GDP-fucose de novo biosynthesis suggests the presence of a novel fucose-containing glycoconjugate in Plasmodium asexual blood stages
Source: Sci Rep. 2016 Nov 16;6:37230. doi: 10.1038/srep37230 (PMC5110956; doi:10.1038/srep37230)
Supplement: Supplementary Information [file srep37230-s1.pdf]

The disruption of GDP-fucose de novo biosynthesis suggests the presence of a novel fucose-containing glycoconjugate in *Plasmodium* asexual blood stages
Sílvia Sanz, Borja López-Gutiérrez, Giulia Bandini, Sebastian Damerow, Sabrina Absalon, Rhoel R. Dinglasan, John Samuelson and Luis Izquierdo

Fig. S1

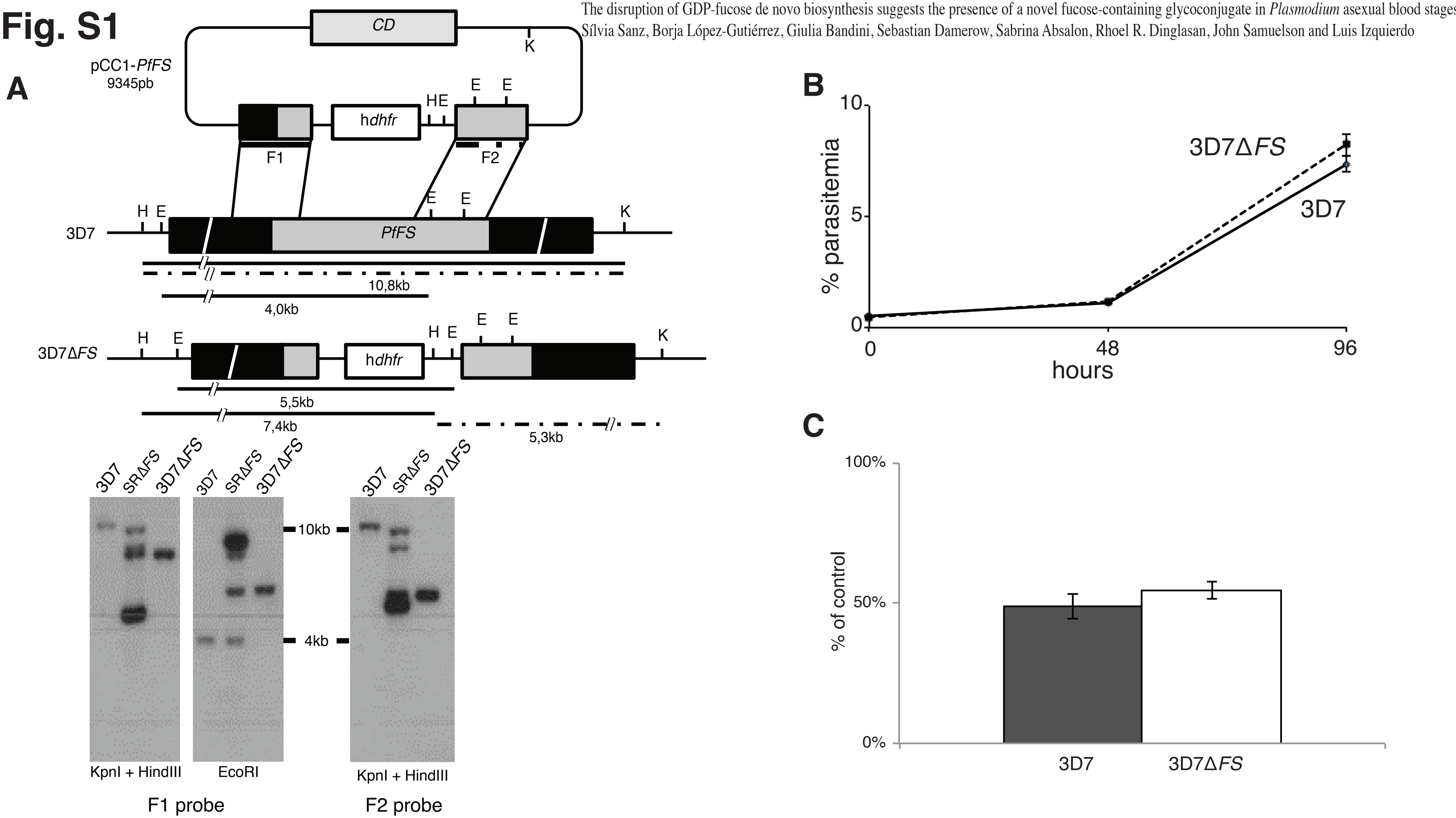

Fig. S1. *P. falciparum* 3D7Δ*FS* mutant generation and phenotyping. (A) (Top) Schematic representation of the transfection plasmid (pCC1-*PfFS*) used to target and disrupt FS gene in *P. falciparum* 3D7 parasites (3D7) and the expected double crossover recombination events (3D7Δ*FS*). The black boxes represent upstream and downstream DNA sequence flanking the *PfFS* gene locus. The position of HindIII (H), KpnI (K) and EcoRI (E) restriction sites, the predicted length of restriction fragments and the position of F1 and F2 probes (black and dotted line respectively) are shown. (Bottom) Southern Blot analysis of EcoRI and KpnI+HindIII digested genomic DNA from 3D7, SRΔFS (single recombinant parasites) and 3D7ΔFS parasites. SR refers to Single recombinant parasites, parasites before negative selection with 5-Fluorocytosine. Hybridisation of F1 and F2 probes to digested DNA from 3D7ΔFS parasites revealed restriction fragment sizes consistent with disruption of *PfFS* by integration of the hdhfr drug-resistance cassette. (B) Synchronous ring-stage 3D7Δ*FS* and 3D7 growth was monitored over two complete life cycles (96h) by flow cytometry. (C) Inhibition of 3D7 (dark grey bar) and 3D7Δ*FS* (white bar) growth by a 3-h heat-shock at 41.5oC. Values are the average of three independent replicas, with standard deviation, and represent percentage of growth relative to identical cultures not subjected to heat-shock. Statistical analysis showed no significant differences between cell lines.

The disruption of GDP-fucose de novo biosynthesis suggests the presence of a novel fucose-containing glycoconjugate in *Plasmodium* asexual blood stages

Sílvia Sanz, Borja López-Gutiérrez, Giulia Bandini, Sebastian Damerow, Sabrina Absalon, Rhoel R. Dinglasan, John Samuelson and Luis Izquierdo

Supplementary TABLE SI

Sugar nucleotide levels in 3D7 (wild type) and 3D7 Δ *GMD* cell lines, measured at the trophozoite stage in two different sets of experiments

Sugar nucleotide [a]	Experiment 1			Experiment 2		
	3D7	3D7Δ*GMD*	Lysed RBCs	3D7	3D7 Δ*GMD*	Lysed RBCs
UDP-Glc	1.87 ± 0.09	0.57 ± 0.02	0.006 ± 0.002	3.07 ± 0.09	3.00 ± 0.06	0.002 ± 0.00 [c]
UDP-Gal	0.39 ± 0.02	0.12 ± 0.01	0.003 ± 0.001	0.71 ± 0.04	0.69 ± 0.03	NQ [b]
UDP-GlcNAc	2.27 ± 0.09	0.66 ± 0.02	0.048 ± 0.016	2.85 ± 0.04	2.88 ± 0.08	0.008 ± 0.002
GDP-Man	0.51 ± 0.01	0.22 ± 0.00 [c]	NQ [b]	1.17 ± 0.02	1.61 ± 0.04	NQ [b]
GDP-Fuc	0.50 ± 0.01	0.22 ± 0.00 [c]	NQ [b]	0.32 ± 0.01	0.32 ± 0.01	NQ [b]

[a] Amounts are indicated in pmoles/10^7 cells

[b] Not Quantified (signal to noise ratio < 10)

[c] SD at least two orders of magnitude below the calculated amount

Fig. S2

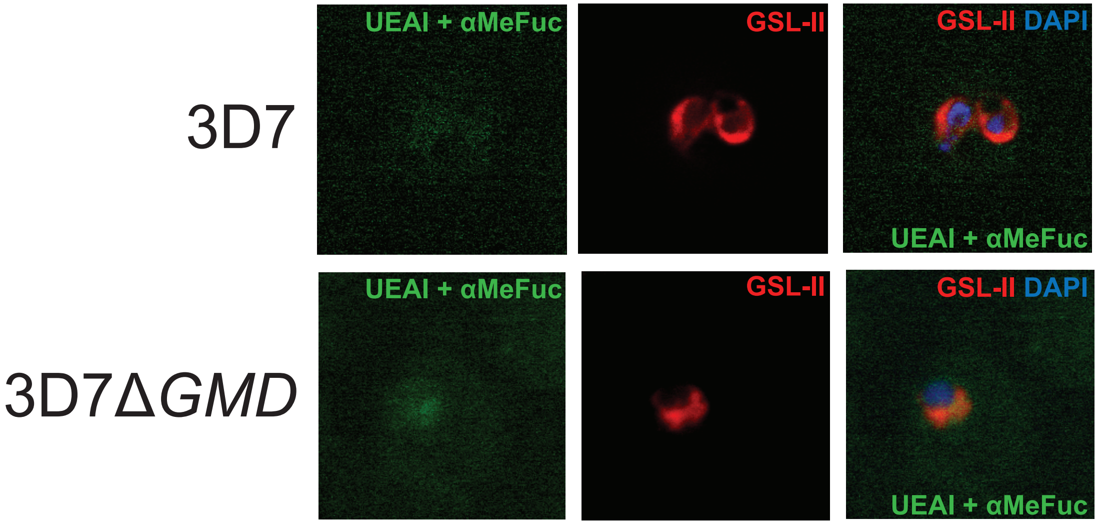

Fig. S2. Deconvolving micrographs of wild type (3D7) and mutant (3D7Δ*GMD*) *P. falciparum* infected RBCs labelled with α MeFuc preincubated UEA-I lectin. Cultured wild type and mutant *P. falciparum* lines were washed in PBS and smeared in glass slides. After drying, the smears were fixed in 4% PFA in PBS for 20 min ar RT. Fixed cells were washed, permeabilised in 0.1% TX-100 in 1xPBS for 5 min at RT, washed again and then blocked in 3% BSA in 1xPBS overnight at 4°C before incubation with UEA-I and GSL-II lectins and DAPI-staining. Preparations were mounted using Vectashield and examined on a Zeiss AXIO inverted microscope with Colibri LED and Hamamutsu Orca-R2 CCD camera. Images were collected at 0.2-µm optical sections and deconvolved using Zen. Images show single z slices.

Fig. S3

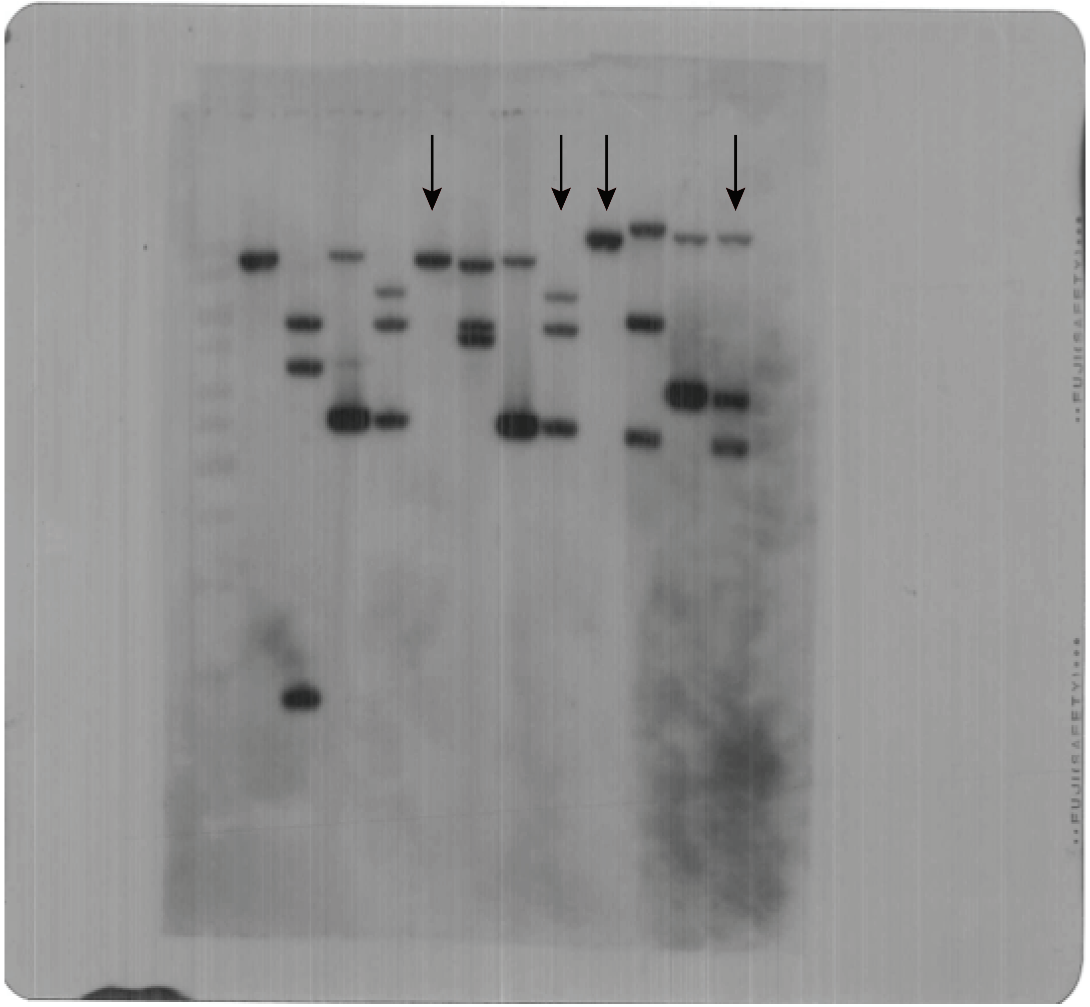

Fig. S3. Full-length Shouthern blot showing the lanes used for Fig. 1B. Arrow marks indicate the selected lanes used to illustrate the generation of 3D7Δ*GMD* cell line. Other lanes include either the use of extra restriction enzymes (not included in Fig. 1B), parasite cell lines where the recombination did not work or cell lines not related to this study
